# Behavioural activation written self-help to improve mood, wellbeing and quality of life in people with dementia supported by informal carers (PROMOTE): a study protocol for a single-arm feasibility study

**DOI:** 10.1186/s40814-016-0083-x

**Published:** 2016-08-04

**Authors:** Paul Farrand, Joanne Woodford, David Llewellyn, Martin Anderson, Shanker Venkatasubramanian, Obioha C. Ukoumunne, Anna Adlam, Chris Dickens

**Affiliations:** 1 Clinical Education Development and Research (CEDAR), Psychology: College of Life and Environmental Sciences, University of Exeter, Washington Singer Labs, Perry Road, Exeter, EX4 4QG UK; 2 University of Exeter Medical School, St. Luke’s Campus, Exeter, EX1 2LU UK; 3 NIHR CLAHRC South West Peninsula, University of Exeter Medical School, St. Luke’s Campus, Exeter, EX1 2LU UK

**Keywords:** Dementia, Depression, Behavioural activation, Caregivers, Feasibility

## Abstract

**Background:**

Increases in life expectancy have resulted in a global rise in dementia prevalence. Dementia is associated with poor wellbeing, low quality of life and increased incidence of mental health difficulties such as low mood or depression. However, currently, there is limited access to evidence-based psychological interventions for people with dementia experiencing low mood and poor wellbeing. Behavioural activation-based self-help, supported by informal carers and guided by mental health professionals, may represent an effective and acceptable solution.

**Methods/design:**

The present study is a phase II (feasibility) single-arm trial informed by the Medical Research Council complex interventions research methods framework. Up to 50 dementia participant/informal carer dyads will be recruited from a variety of settings including primary care, dementia-specific health settings and community outreach. People living with dementia will receive behavioural activation-based self-help and be supported by their informal carer who has received training in the skills required to support the self-help approach. In turn, during the use of the intervention, the informal carer will be guided by mental health professionals to help them work through the materials and problem solve any difficulties. Consistent with the objectives of feasibility studies, outcomes relating to recruitment from different settings, employment of different recruitment methods, attrition, data collection procedures, clinical delivery and acceptability of the intervention will be examined. Clinical outcomes for people with dementia (symptoms of depression and quality of life) and informal carers (symptoms of depression and anxiety, carer burden and quality of life) will be measured pre-treatment and at 3 months post-treatment allocation.

**Discussion:**

This study will examine the feasibility and acceptability of a novel behavioural activation-based self-help intervention designed to promote wellbeing and improve low mood in people living with dementia, alongside methodological and procedural uncertainties associated with research-related procedures. As determined by pre-specified progression criteria, if research procedures and the new intervention demonstrate feasibility and acceptability, results will then be used to inform the design of a pilot randomised controlled trial (RCT) to specifically examine remaining methodological uncertainties associated with recruitment into a randomised controlled design.

**Trial registration:**

Current Controlled Trials ISRCTN42017211

## Background

Dementia is a global healthcare concern, with 115.4 million people worldwide expected to be living with dementia by 2050 [1]. Given there are no current cure or preventative medical interventions [2], dementia represents a significant challenge for health policy [3]. Current estimates indicate that on a global scale, prevalence stands at 35.6 million with 670,000 living with dementia in the United Kingdom (UK) [4]. The provision of long-term support to help people with dementia ‘live well’ is, therefore, a global health and social care priority [5–7].

Developing approaches to facilitate long-term support is especially important given quality of life, increased levels of mortality, increased health and social care costs [8] and poorer functional outcomes [9] commonly experienced by people with dementia [10]. Furthermore, between 30 % [11, 12] and 50 % [13] of people with dementia also experience elevated symptoms of depression. However, despite depression being one of the most common mental health difficulties experienced by people living with dementia [14], access to evidence-based psychological therapies remains limited [15]. This treatment gap [16] exists despite growing evidence identifying cognitive behavioural therapy (CBT) as an effective intervention for treating depression in people with dementia [12], partly due to costs of delivery and a lack of trained therapists [17–19].

To provide a potential solution to address this treatment gap, CBT provided in a self-help format is being introduced into mental health services on a global scale [20–23]. CBT self-help is defined as CBT-specific therapeutic techniques being communicated in the form of bibliotherapy, online, audio or smartphone applications [24, 25] as opposed to delivery by a therapist [24, 26]. Some evidence suggests effectiveness increases when some form of face-to-face, telephone, or email guidance or support is also provided [26–28]. Within the Improving Access to Psychological Therapies (IAPT) Programme implemented across England [29], support is provided by a practitioner-based workforce (Psychological Wellbeing Practitioners (PWPs)) and introduced alongside face-to-face ‘high-intensity’ evidence-based psychological therapies within a stepped care model of delivery [30]. Recent evidence also suggests support can be provided by non-professionals [31]. As such, a potential solution to increase access to psychological therapies for people with dementia may be for non-professionals, such as informal carers defined as those providing unpaid and untrained support within the community to a care recipient [32], to support therapy delivery. The provision of informal carer itself is associated with increased mental health difficulties such as anxiety and depression [33], restriction of social and recreational activities [34] and poor quality of life [35]. Conversely however, other evidence suggests that involving informal carers in the facilitation of psychological interventions for people living with dementia may also improve caregiver mood [36].

Behavioural activation (BA) is a psychological intervention featuring prominently within the IAPT programme [37] and is an evidence-based and cost-effective [38, 39] treatment for depression. BA aims to overcome depression through a structured and graded approach to reintroduce activity into people’s lives to target behavioural avoidance [37] which is common in depression. Specifically, techniques used in BA help reintroduce people to sources of positive-reinforcement within their environment, whilst overcoming sources of negative reinforcement that maintain avoidance behaviours [40]. Several characteristics of the BA self-help protocol utilised within the IAPT programme [40] suggest it may also have potential utility for people with dementia. Not only is BA considered a straightforward approach making it easier for users with dementia and their carers to understand [41] but it may also complement the range of self-management techniques people with dementia and carer dyads already utilise [42]. Initial evidence has already highlighted the potential utility of BA as an effective intervention for treatment of depression in people with Alzheimer’s disease, supported by a therapist and informal carer [36, 43]. However, these studies focused on the use of experienced geriatricians to deliver face-to-face therapy [36, 43], analogous to CBT ‘high-intensity’ support within a stepped care model of delivery [30]. Implementing this ‘high-intensity’ intervention delivered face-to-face by an experienced geriatrician workforce, who may already face immense demands on their time with other aspects of their role may, therefore, be prohibitive and unable to meet the potential demand for treatment [44].

A research programme informed by the Medical Research Council (MRC) complex interventions framework [45] has been undertaken to develop a written BA-based self-help intervention to target low mood and improve wellbeing in people with dementia, supported by their informal carers who themselves are guided in delivering the intervention by a practitioner-based PWP workforce. The current feasibility study builds upon MRC phase I development work [45] previously completed. Recognising the importance of involving people living with dementia and their carers actively in research [46], the phase I study involved semi-structured interviews with people with dementia to develop a new written BA-based self-help intervention to meet the needs of people with dementia. Additionally, two focus groups took place with carers about their role in supporting the person living with dementia work through the materials and problem solve any difficulties encountered under PWP guidance. This protocol represents MRC phase II (feasibility) research [45] to examine methodological, procedural and clinical uncertainties [47, 48]. Should progression criteria for this feasibility study be met, results will inform the design and funding application for a further phase II (pilot) randomised controlled trial (RCT) that will examine methodological and procedural uncertainties specifically related to an RCT design. Subsequent progression of this research programme to a possible future definitive (phase III) RCT will be informed by results from the piloting phase.

### Study aims and objectives

A single-arm feasibility phase II study [45] with an embedded qualitative component examining a number of feasibility questions pertaining to methodological, procedural and clinical uncertainties [47, 48] will be conducted.

The following outcomes will be examined following guidance concerning feasibility study objectives, as distinguished from objectives for a pilot RCT [47]:

1. The intensity of the recruitment procedure in terms of number of invitation packages sent by health professionals
2. Number of health professionals required to recruit into the study
3. The time taken (up to 6 months) to recruit the a priori determined sample size
4. Willingness of clinicians to recruit participants across multiple recruitment settings utilising different recruitment strategies
5. Participant response rates between different recruitment techniques
6. Eligibility proportions
7. Participant level barriers to recruitment.
8. Study resources required to implement study procedures
9. Feasibility and acceptability of data collection procedures to participants
10. Feasibility and acceptability of the intervention to participants and clinicians
11. Barriers to clinical delivery
12. Clinician training needs
13. Clinician adherence to the intervention protocol

As determined by pre-specified progression criteria, outcomes will be used to inform the design of a subsequent pilot RCT.

## Methods/design

### Study design

A feasibility phase II single-arm trial [45] with an embedded qualitative sub-study will be conducted. This protocol (version 1, 18/08/2015) is registered on Current Controlled Trials ISRCTN42017211 and follows Standard Protocol Items, Recommendations for Interventional Trial (SPIRIT) [49] guidelines for reporting interventional trials.

### Setting

Four different settings—general practitioners (GPs), specialist dementia healthcare settings employing Primary Care Dementia Practitioners (PCDP), memory clinics and community outreach in the county of Cornwall (southwest England)—will be utilised to examine feasibility outcomes associated with recruitment. All treatment will be provided by PWPs within ‘step 2’ of an IAPT primary care mental health service adopting a stepped care model [30]. Recruitment setting locations have been selected on the basis of locality of study PWPs from the IAPT service to help increase the feasibility of intervention delivery. Further, recruitment setting types were selected on the basis of recruitment strategies utilised in other depression [50–52] and dementia studies [53, 54].

### Eligibility criteria

#### People living with dementia

Participants are included if they:

1. Have a diagnosis of probable dementia recorded in medical records, with no restriction placed on dementia type given evidence suggesting mixed dementia is the most common dementia presentation [55]
2. Have mild-to-moderate dementia severity defined as scoring between 12 and 24 on the mini-mental state examination (MMSE) [56]
3. Have a score of 4 or more on the 12-item Geriatric Depression Scale (GDS-12R) [57]
4. Residing at home
5. Able to provide informed consent or have an informal carer willing to provide consultee consent
6. Have sufficient proficiency in English to read and engage with the BA-based self-help material
7. Have an informal carer (defined as a partner, family member or friend) who has regular contact (at least weekly) with the person with dementia and willing to support the intervention.

Participants receiving antidepressant medication and/or acetyl-cholinesterase inhibitors or memantine will also be able to participate if they have been receiving a stable dose for at least 1 month before recruitment. No upper or lower age restrictions are placed on people living with dementia.

Participants are excluded if they:

1. Have a co-morbid diagnosis of a severe and enduring mental health problem including psychosis, type I or II bipolar disorder and personality disorder recorded in medical records or self-reported to the study team
2. Are currently receiving formal psychotherapy or another potentially active psychological treatment
3. Are acutely suicidal and/or have a history of persistent self-injury
4. Have self-report or medical record documented misuse of alcohol, prescription drugs or street drugs, so severe it interferes with the person with dementia’s ability to perform normal activities in daily life and engagement with the intervention

Exclusions 1–3 are informed by guidance indicating that CBT self-help is not suitable for the specific population identified or that people with depression should only receive one psychological intervention at a time [58].

#### Informal carers

Participants are included if they are:

1. Aged 16 years or over.
2. Self-identified informal carer of a person with dementia with regular contact (at least weekly) with the person with dementia
3. Willing to support the intervention, including increasing contact to facilitate supporting the intervention if required

Participants are excluded if they:

1. Score over 20 on the Patient Health Questionnaire-9 (PHQ-9) indicating severe levels of depression [59]
2. Have a co-morbid diagnosis of a severe and enduring mental health problem including post-traumatic stress disorder (PTSD), psychosis, type I and II bipolar disorder or personality disorder as recorded in medical records or self-reported to the study team
3. Have self-report or medical record documented misuse of alcohol, prescription drugs or street drugs, so severe it interferes with the informal carer’s ability to perform normal activities in daily life and engagement with the intervention
4. Have difficulties reading or following the written BA self-help material
5. Are acutely suicidal and/or have a history of persistent self-injury

The exclusion criteria for informal carers are based upon clinical factors that may impair and interfere with an informal carer’s ability to support and engage in the intervention and indicate the carer themselves may require psychological support.

To be included within the study, both members of the dyad (person with dementia and informal carer) need to meet the inclusion criteria. In the case of a person with dementia being eligible for inclusion, but an informal carer ineligible, the researcher will work with the dyad to identify if another informal carer known to the dyad may be willing to participate in the study.

### Recruitment settings and procedure

**Fig. 1:**
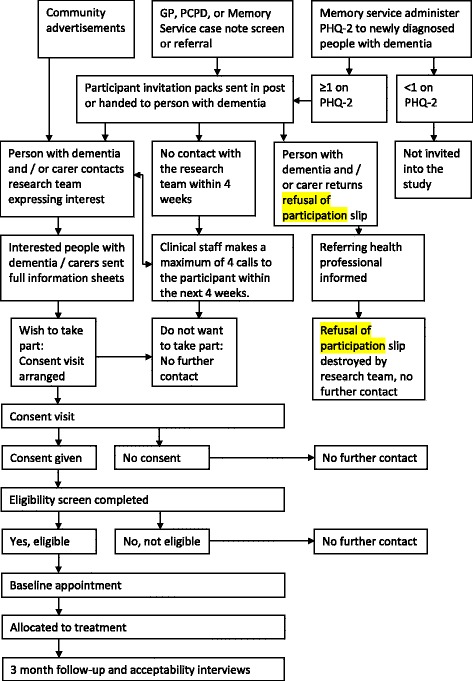
Flow of participants through the study

A multifaceted recruitment approach will be employed, building on techniques used to successfully recruit carers of people with dementia [54] across four specific recruitment settings (see Fig. 1).

#### GP records

GP search and mail-out will be adopted as used successfully in a number of depression trials [50–52]. GP electronic case records will be searched for people with a formal diagnosis of dementia, and a manual screen will be performed to check against the inclusion criteria. Reasons for exclusion will be anonymised and provided to the research team [60]. Participant invitation packages (study invitation letter, study summary sheet, reply slip and reasons for refusal of participation questionnaire) will be sent to people with dementia included in the screen. This package will also include a separate study invitation package to be passed onto an informal carer. People with dementia, or informal carers, will be able to contact the research team by returning the reply slip, telephoning or emailing the research team. Given that telephone reminders by clinicians have been reported to increase recruitment rates [61], non-responders will be provided with telephone follow-up calls by general practice staff. Where phone calls receive no answer, a maximum of four attempts to establish contact will be made over this time period. GPs can also directly refer suitable people with dementia. Study posters and brochures will also be displayed in practice receptions to further advertise the study.

#### Primary care dementia practitioners

PCDPs will perform a search and mail-out. Drawn from a variety of backgrounds in health care, PCDPs are health professionals specialising in dementia care providing support to people living with dementia and their families in the community. PCDPs will screen caseloads to check against the inclusion criteria. Reasons for exclusion will be anonymised and provided to the research team. Study invitation packages will be sent in a manner consistent with that used for GP record recruitment or handed to potential participants face-to-face. It is anticipated that PCDPs will complete their initial search and mail-out within 1 month of the study commencing recruitment with any patients added to their caseload over the 6-month recruitment period being invited face-to-face. PCDPs will provide non-responders with telephone reminders (maximum of four attempts) and can also directly refer suitable people with dementia during the course of the study.

#### Memory service

The memory service (Consultant in Old Age Psychiatry and Memory Assessment Nurses) will recruit potential participants using two methods: (1) post-diagnostic assessments with memory service staff administering the PHQ-2, a two-item screen for possible depression [62, 63], with people with dementia screening positive for possible depression provided with a study invitation package and (2) a search and screening of the memory service database for people who have received a diagnosis of dementia over the preceding 24 months with those identified as potentially eligible being sent the study invitation package in the post. In the case of post-diagnostic assessments only, the administration of the PHQ-2 screen is included within the recruitment procedure to help minimise the number of people invited into the study who do not experience low mood or depression. Post-diagnostic assessments are conducted by health professionals experienced in screening for depression (Consultant in Old Age Psychiatry and Memory Assessment Nurses) and are the only consistent face-to-face recruitment strategy utilised in the study. As such, administration of the PHQ-2 screen will only take place in this recruitment centre. Other face-to-face recruitment techniques (e.g., GP or PCDP referral) are designed to be more opportunistic and time limited, as such the addition of a PHQ-2 screen was not deemed necessary. Given that depression status may have changed since post-diagnostic assessment, people with a diagnosis of dementia recorded on the memory service database will be invited to participate in the study whether or not they have a record of depression documented. Memory service staff will provide non-responders with telephone reminders (maximum of four attempts). Anonymised reasons for exclusion from both the post-diagnostic assessments and database screen will be provided to the research team.

#### Community outreach

A number of community-based organisations including Memory Cafés, Memory Matters South West, the Alzheimer’s Society, Carers Forum, Disability Cornwall, Dementia Action Alliance, Age UK, Cornwall Carers and Penwith Community Development Trust will be approached to support the study by distributing flyers and invitation packages and displaying posters. In addition, the study will be advertised by distributing study posters and leaflets in other community locations including community day care centres; libraries, banks, bus stops, post offices, supermarkets, cafes and community centres.

#### Reasons for refusal of participation

Across all recruitment settings, study invitation packages will include refusal of participation forms for both the person with dementia and informal carer for those who do not wish to participate. The form includes a questionnaire listing possible reasons for refusal of participation informed by previous research [64] as well as an open-ended question to provide further reasons if wished. Each package also includes a freepost envelope to enable people with dementia and their carers to return refusal of participation forms to the study team. These data will be used to inform barriers of recruitment and the acceptability of the proposed intervention.

### Informed consent, screening and baseline

A researcher (MA or SV) will speak to all people with dementia and informal carers who respond to the study invitation package or study advertisements expressing interest to participate or seeking more information about the study. A researcher will arrange to visit those people with dementia and informal carers who are interested in participating in the study, face-to-face, to obtain written consent. Following Mental Capacity Act guidance [65], the researchers will assess capacity to consent in people with dementia, whilst providing appropriate support to maximise their ability to provide consent [66]. If the person with dementia is assessed as lacking capacity to consent, an informal carer will be asked to act as a consultee [65, 67]. Once consent is obtained from the informal carer, they, alongside the person with dementia, will undergo a face-to-face screening assessment with a researcher, in a location identified by the dyad as convenient. If eligibility is confirmed for both, a full baseline assessment will be arranged with the dyad, again in a convenient location. After the full baseline assessment has been conducted, dyads will be allocated to a PWP by a member of the research team, ensuring as best as possible an even balance across PWPs (see Fig. 1).

### Sample size

A primary aim of this study is to examine the recruitment of participants, through a variety of techniques; therefore, the study will not stipulate a sample size a priori. Instead, to facilitate comparisons between the effectiveness of the recruitment approaches adopted, the study will continue to recruit until a maximum cut-off of 50 dyads (100 participants). Fifty dyads will enable the estimation of a follow-up rate of 80 % with a margin of error of 14 % based on the lower bound of the 95 % confidence interval.

### Intervention

#### Content

The BA intervention is informed by the simple BA protocol [38, 40] originally developed for PWP delivery of low-intensity treatment within the IAPT programme [68].

#### Materials

Two workbooks have been developed, one designed for the person with dementia [69] and the other for the informal carer to aid supporting the person with dementia work through the intervention [70]. The workbook for the person with dementia was written in line with national guidance for the development of dementia-friendly written information [71] describing steps of the BA intervention and providing accompanying worksheets. The informal carer workbook provides guidance on how to support the person with dementia implement the steps involved within the BA protocol, alongside additional support to manage in the caring role, informed by an existing self-help intervention for carers of stroke survivors [72]. To maximise acceptability, the content and design of both workbooks were further informed by a series of qualitative studies with people with dementia and their informal carers.

#### Support

A PWP will guide the use of the BA self-help programme, providing a maximum of 12 sessions over 3 months (see Table 1 for the treatment support protocol). These 12 sessions will include one initial assessment session, one setting up support session, up to nine brief telephone support ‘check-ins’ representing minimal contact support [26, 73] to the informal carer and finally, one relapse prevention session. PWPs will follow a structured support protocol, adapted from existing support protocols for PWP assessment and support sessions [68] and brief telephone support sessions [75].

**Table 1:** Treatment support protocol

Session number	Attendees	Method of support	Session content	Session duration (min)
1	Person with dementia; informal carer; PWP	Face-to-face	Problem focused assessment to identify the main difficulties with mood and wellbeing experienced by the person with dementia and to introduce the BA approach. The informal carer will also be present to act as an informant if required.	50
2	Person with dementia; informal carer; PWP	Face-to-face	A setting up support session to help establish the protocol for supporting the BA self-help intervention. The rationale for the BA intervention is discussed alongside the procedure for the carer to provide on-going support to the person with dementia.	40
3–11 (maximum)	Informal carer; PWP	Telephone	Minimal contact telephone ‘check-ins’ to check progress made with the intervention, problem solve any difficulties experienced with using the workbook, agree next steps and provide on-going encouragement in the use of the BA self-help workbooks.	Up to 15
12	Person with dementia; informal carer; PWP	Face-to-face	Relapse prevention and provision of information to enhance on-going signposting to appropriate health and social care organisations as needed.	40

#### Setting

All participants will be treated within the ‘BeMe’ primary care mental health service commissioned under the IAPT programme [29] and part of Cornwall Partnership NHS Foundation Trust. All face-to-face sessions will be offered in BeMe offices, community settings or the participants’ home if necessary to ensure inclusion into the study for all those wishing to participate.

#### PWPs

PWPs will be existing employees of the IAPT primary care mental health service supporting the study. Initially, three existing staff members will be asked to support the study by the service clinical lead, with additional PWPs trained to support the study should capacity need to be increased. All PWPs supporting the intervention will have to successfully complete the IAPT PWP training programme [68] to support the delivery of CBT self-help interventions. Additionally, a 2-day training session specific to supporting the new intervention will be delivered. Group-based clinical supervision to all PWPs supporting the intervention will be provided once a month by the principal investigator, PF.

#### PWP adherence

With participant consent, all assessment and support sessions will be recorded. To assess adherence to the support protocol, the marking criteria used as the basis of competency assessments within the IAPT training programme for PWPs will be adopted [68]. Necessary adaptations to the support protocol and associated marking criteria have been undertaken to ensure the low-intensity CBT clinical method is consistent and appropriate for use in the study with people with dementia and informal carers. A sample of 20 % of sessions will be randomly selected for each PWP, and adherence to the support protocol will be judged by a clinician otherwise not associated with the study. This clinician will be considered competent to mark the tapes by virtue of having completed and passed the IAPT programme for the training for PWPs themselves and having undertaken additional training with the research team in the use of the marking criteria and adaptations for a dementia population.

### Feasibility outcome measurements (primary outcome measurements)

Data related to study feasibility will be collected to examine the primary study objectives relating to methodological, procedural and clinical uncertainties [47, 48]. Following guidance concerning feasibility study objectives [47], Table 2 provides a summary of feasibility outcomes to be examined, alongside progression criteria (where applicable) to be met in order to progress to develop an application for phase II pilot RCT funding [45].

**Table 2:** Feasibility data and method of measurement

Feasibility outcome	Measurement	Progression criteria to phase II pilot RCT
Recruitment	Quantitative data	
	Percentage of people with dementia invited into the study/number of people with dementia in total identified by health professionals	No criteria set
	Number of health professionals required to assist with recruitment into the study	No criteria set
	The time taken (up to 6 months) to recruit up to 50 dyads	No criteria set
	Number of dyads enrolled into the study per week	2 dyads per week
	Percentage of dyads willing to undergo screening/number invited (calculated for GP, PCPD and memory service recruitment)	≥15 %
	Percentage of dyads overall meeting the inclusion criteria/number invited	≥5 %
	Percentage of dyads overall enrolled in the study/number invited	≥5 %
	Qualitative data	
	Reasons for exclusion reported to the research team during health professional screening (GP, PCPDs, memory service)	No criteria set
	Reasons for ineligibility	No criteria set
	Identified barriers to recruitment (reasons for refusal of participation)	No criteria set
Attrition	Quantitative data	
	Percentage of dyads completing post-treatment (3 month) outcome measures	≥70 %
	Reasons for dropout	No criteria set
	Qualitative data	
	Acceptability interviews with non-attendees and poor attendees (informal carers and people with dementia)	No criteria set
Data collection procedures	Quantitative data	
	Time taken and number of sessions to administer the screening measures	≤2 h; ≤2 sessions
	Time taken and number of sessions to administer the baseline assessment	≤2 h; ≤2 sessions
	Time taken and number of sessions to administer the follow-up assessments	≤2 h; ≤2 sessions
	Percentage of missing items per questionnaire	≤10 %
	Qualitative data	
	Acceptability interviews with participants concerning acceptability of research procedures	No criteria set
	Acceptability interviews with PWPs concerning acceptability and feasibility of research procedures	No criteria set
Clinician adherence	Adherence to support protocol as determined by therapy tapes	≥70 %
Clinical delivery	Quantitative data	
	Time between being allocated to PWP and PWP undertaking the assessment session.	≤2 weeks
	Session lengths	No criteria set
	Number of sessions received per dyad	No criteria set
	Settings of sessions (e.g., BeMe, community, home)	No criteria set
	Number of missed appointments	No criteria set
	Number of missed outcome measurement items	No criteria set
	PWP attrition	No criteria set
	Impact of severity of dementia (MMSE score) informing who can engage in the intervention	No criteria set
	Qualitative data	
	Acceptability interviews with participants	No criteria set
	Acceptability interviews with PWPs	No criteria set

### Clinical outcome measurements (secondary outcome measurements)

To examine the feasibility of the data collection procedures, a number of clinical outcome measures will be collected from people with dementia and their informal carers (see Table 3).

**Table 3:** Study clinical outcome measurements by time point

Outcome measure	Time point
Person with dementia	
Sociodemographics	Initial screen
MMSE [56]	Initial screen
GDS-12R [57]	Initial screen, post-treatment follow-up
CSDD [76]	Baseline, post-treatment follow-up
DEMQOL [81]	Baseline, post-treatment follow-up
EQ-5D-3L [82]	Baseline, post-treatment follow-up
Informal carer	
Sociodemographics	Initial screen
PHQ-9 [84]	Initial screen, post-treatment follow-up
GAD-7 [85]	Baseline, post-treatment follow-up
ZBI-12 [86]	Baseline, post-treatment follow-up
CSDD-proxy [76]	Baseline, post-treatment follow-up
SF-12 [87]	Baseline, post-treatment follow-up
EQ-5D-3L [83]	Baseline, post-treatment follow-up
Revised CSRI [88]	Baseline, post-treatment follow-up

*MMSE* mini-mental state examination, *GDS-12R* Geriatric Depression Scale-12 Residential, *CSDD* Cornell Scale for Depression in Dementia, *DEMQOL* Dementia Quality of Life Measure, *EQ-5D-3L* EuroQol-5D-3L, *PHQ-9*, Health Questionnaire-9, *GAD-7* Generalised Anxiety Disorder 7-Item Scale, ZBI-12 Zarit Caregiver Burden Interview Short Form, *SF-12* 12-Item Short Form Health Survey, *CSRI* Client Service Receipt Inventory

#### People with dementia

The Cornell Scale for Depression in Dementia (CSDD) [76] will be used to assess severity of depressive symptoms, alongside the GDS-12R [48]. The CSDD [71] will be used to assess severity of depressive symptoms, alongside the GDS-12R [57, 77]. The CSDD is an interview-based questionnaire conducted with both the person with dementia and an informal carer as a proxy measure and demonstrated to be a reliable and valid measure for people with both mild and moderate-to-severe levels of dementia [78, 79]. The GD-12R is reliable for people with moderate-to-severe levels of dementia [57, 77] and recommended for use in psychosocial intervention research for people with dementia [80]. Quality of life will be examined through the Dementia Quality of Life measure [81] and the EuroQol-5D-3L (EQ-5D-3L) [82] validated in a mild-to-moderate dementia population [83].

#### Informal carers

The PHQ-9 [84] and the Generalised Anxiety Disorder Questionnaire (GAD-7) [85] will be administered to assess severity of depressive and anxious symptoms, respectively. Carer burden will be measured through the administration of the Zarit Caregiver Burden Interview Short Form (ZBI-12) [86]. Quality of life will be assessed through the Medical Outcome Survey Short Form 12 (SF-12) [87] and EQ-5D-3L [82]. Health and social care use, for both the person with dementia and informal carer, will be collected via the administration of an adapted version of the Client Socio-Demographic and Service Receipt Inventory (CSRI) [88], based on two versions developed for carers of stroke survivors [74, 89]. In addition, the CSDD-proxy [76] will be administered to informal carers to collect further information concerning the severity of depressive symptoms in the person with dementia.

#### Sociodemographics and clinical characteristics

During the initial screen, various background, clinical and socio-demographic characteristics—age, gender, ethnic background, relationship status, employment status, highest level of academic qualification, yearly household income, chronic physical health conditions—will be collected from people with dementia and informal carers. This will be supplemented by the additional collection of dementia subtype diagnosed and date of diagnosis from the person with dementia. Informal carers will also be asked to confirm the dementia subtype diagnosed and date of diagnosis as well as yearly household income (if the carer does not live with the person with dementia), length of time caring, if the carer lives with the person with dementia, provision of care before diagnosis of dementia, receipt of support services in the home, hours of support services received in the home per week and hours of caring per week.

### Data collection

Researchers will collect data from the person with dementia and informal carer face-to-face, at a location convenient for the person with dementia and informal carer. To protect confidentiality, each dyad will be offered the choice of completing outcome measurements at an alternative face-to-face appointment to the other member of the dyad should they prefer. Data will be collected at screening, baseline, and post-treatment (3 months post-treatment allocation). Clinical outcome measurements collected at each time point are summarised in Table 3.

### Intervention acceptability

#### Study objectives and design

An embedded qualitative study will be conducted to determine the views of people with dementia and informal carers regarding the acceptability of the BA self-help intervention. PWPs supporting the intervention will be interviewed with respect to the acceptability and feasibility of the BA self-help intervention and support protocol. All people with dementia and informal carers will be invited to participate in semi-structured face-to-face or telephone-based interviews using open-ended questions addressing:

1. Relevance of the intervention
2. Suitability of the intervention
3. Impressions of guidance provided by PWPs
4. Perceived benefit of the intervention
5. Problems experienced utilising the intervention
6. Continued use of the intervention
7. Recommendations for further treatment development
8. Acceptability of research processes

The interview topic guide is informed by interview topic guides for CBT self-help for patients with multiple sclerosis [90] and carers of stroke survivors [74]. Informal carers and those with dementia who have poor session attendance will be invited to take part in an interview session which will ask questions concerning the reasons for disengaging with the intervention and recommendations for a more acceptable intervention. Interviews are estimated to last between 30 and 60 min, with shorter times expected for non/poor attendees.

Semi-structured interviews with PWPs will be conducted over the telephone and are expected to last between 45 and 60 min. Open-ended questions will be asked about the PWP’s impressions of the following:

1. The self-help intervention
2. Difficulties or problems encountered providing support
3. Recommendations for future development of the intervention and PWP training
4. Acceptability and feasibility of collecting in-session outcome measurements
5. Acceptability and feasibility of research processes

#### Sampling

All participants will be invited to participate in the acceptability interviews. The research team will attempt to interview participants categorised into one of the following groups: (1) non-attendees (no sessions attended); (2) poor attendees (participants who attend the assessment session but terminate treatment prior to making a collaborative decision with the PWP to stop treatment); or (3) completers (engagement in treatment until a collaborative decision with the PWP to stop treatment is made). All study PWPs will be interviewed concerning the acceptability of the intervention.

### Statistical analysis

#### Quantitative

Recruitment data: where possible, data will be collected separately from each recruitment setting to determine recruitment rate and methodological uncertainties associated with each specific setting. Data will be collected relating to the type and number of referring sites; number of participants identified at each site; number of invites sent (where possible, this will not be possible with community recruitment), number of potential participants requesting more information, number of consents obtained, number of screens completed, number of baselines completed and number of participants allocated to a PWP. Number of exclusions at each stage and reasons for exclusions will also be reported. The percentage of eligible participants that are recruited will be calculated with exact 95 % confidence intervals using the cii command in Stata software version 14.0.

Feasibility of data collection procedures and acceptability to patients: study protocol deviations along with reasons will be reported to assess both the feasibility and acceptability of the data collection process. The time taken to administer the screening measures, time taken to administer the baseline assessment, time taken to administer the follow-up assessments and the percentage of missing data per outcome measurement collected will also be reported to examine the feasibility and acceptability of data collection procedures.

Attrition (study dropout): the number of participants dropping out of the study will be reported, along with the stage of dropout and reasons where possible. Attrition proportions will be reported with 95 % confidence intervals.

Primary and secondary outcome measurements: descriptive statistics including the means and standard deviations or medians and interquartile ranges will be reported for each outcome measurement at baseline and 3 months post-treatment allocation as these will help to inform the sample size for the pilot RCT and later definitive trial.

#### Qualitative

A thematic analysis approach [91] will be adopted to analyse transcribed digital recordings from the interviews. To ensure rigour [92, 93] and confirm consistency with generated themes, two members of the research team (MA, SV) will analyse each interview separately with analyses then compared. Two other research team members (JW and PF), alongside members of the lived experience steering committee (details below), will discuss a subset of the analysed interviews to additionally ensure the analysis reflects the generated themes.

### Lived experience steering committee

A lived experience steering committee has been established as research collaborators [94], consisting of two people with dementia and two informal carers (one spouse and one adult child carer of their mother). Specifically, the lived experience steering committee is responsible for assisting with research activities such as developing accessible participant materials, advising on recruitment and research procedures and supporting PWP training and analysis of the qualitative data. The steering committee will aim to meet monthly throughout the course of the study as active members of the wider research team.

### Dissemination

Findings of the study will be published in an open access journal and via conference presentation. The data from this study will be used to inform the design and accompanying grant application for a future pilot RCT should the study be considered feasible with set progression criteria met.

## Discussion

To the best of our knowledge, this feasibility study represents the first investigation into the feasibility and acceptability of a written BA-based self-help intervention for the treatment of depression in people with dementia. A BA self-help intervention, guided by PWPs and supported by informal carers has the potential to represent an effective, acceptable and cost-effective solution to address the significant unmet need in the provision of psychological support for people with dementia. Furthermore, equipping informal carers with strategies to help improve the wellbeing of care recipients may also improve mood and reduce burden in the carers themselves [36]. This current study will explore important questions pertaining to the acceptability and feasibility of the new intervention and research procedures.

Should the current study demonstrate feasibility and achieve the progression criteria specified, outcomes will be used to design and inform a funding application for a phase II pilot RCT [45]. The aim of this pilot RCT will be to specifically test trial processes associated with an RCT design, with results used to develop and inform a funding application for a future definitive phase III [45] trial to examine effectiveness.

### Study status

Study recruitment will commence in January 2016. The final outcome data will be collected in September/October 2016.

## Abbreviations

BA, behavioural activation; CSRI, Client Socio-Demographic and Service Receipt Inventory; CBT, cognitive behavioural therapy; CSDD, Cornell Scale for Depression in Dementia; GAD-7, Generalised Anxiety Disorder Questionnaire; GDS, Geriatric Depression Scale; IAPT, Improving Access to Psychological Therapies; MRC, Medical Research Council; MMSE, mini-mental state examination; PHQ-9, Patient Health Questionnaire-9; PWP, Psychological Wellbeing Practitioner; RCT, randomised controlled trial; SF-12, Medical Outcome Survey Short Form; SPIRIT, Standard Protocol Items, Recommendations for Interventional Trials; ZBI-12, Zarit Caregiver Burden Interview Short Form
